# Characteristics and Transformation Mechanism of Nonmetallic Inclusions in 304 Stainless Steel during Heat Treatment at 1250 °C

**DOI:** 10.3390/ma13235396

**Published:** 2020-11-27

**Authors:** Wen-Sheng Yang, Shuai Liu, Shao-Wei Han, Jia-Wei Wang, Jing Guo, Yan Yan, Han-Jie Guo

**Affiliations:** 1 School of Metallurgical and Ecological Engineering, University of Science and Technology Beijing (USTB), Beijing 100083, China; yws3179608@163.com (W.-S.Y.); lsls1226@163.com (S.L.); b20160095@xs.ustb.edu.cn (Y.Y.); guohanjie@ustb.edu.cn (H.-J.G.); 2 Steelmaking Department, Beijing Shougang Co., Ltd, Qian’an 064400, China; hanshaowei2016@163.com; 3 Material Research and Surface Engineering Research Center, Dongfang Electric Corporation Dongfang Turbine Co., LTD, Deyang 618000, China; wangjiaweistudy@gmail.com

**Keywords:** nonmetallic inclusion, heat treatment process, Ostwald ripening, stainless steel

## Abstract

Evolutions of two typical types of nonmetallic inclusions, i.e., inclusions based on CaO-SiO_2_-Al_2_O_3_ and MnO-SiO_2_-Al_2_O_3_ of 304 stainless steel were investigated in laboratory-scale experiments under isothermal heat treatment at 1250 °C for 0, 30, 60 and 120 min. Results show inclusion population density increases at the first stage and then decreases while their average size decreases and then increases. Moreover, almost no Cr_2_O_3_ content within the inclusion before the heat treatment, but Cr_2_O_3_ content increases gradually along with increasing heat treatment time. Furthermore, the increasing of Cr_2_O_3_ content in the inclusions would increase their melting points and reduce their plasticities. The experimental results and thermodynamic analysis indicate that there are three steps for inclusion evolution during the heat treatment process, in which Ostwald ripening plays an important role in inclusion evolution, i.e., inclusions grow by absorbing the newly formed small-size MnO-Cr_2_O_3_ inclusions.

## 1. Introduction

There are two main types of no-inclusions in Si-killed stainless steel are CaO-SiO_2_-Al_2_O_3_-based and MnO-SiO_2_-Al_2_O_3_-based systems [1,2,3,4,5]. They have different properties and different effects on the surface quality of stainless steel strips. Researchers [1,2,3,4,5,6,7,8,9,10] found that an effective way to improve the surface quality of stainless steel strip by controlling the nonmetallic inclusion as plasticity MnO-SiO_2_-Al_2_O_3_-based inclusion instead of CaO-SiO_2_-Al_2_O_3_-based ones. This could be realized by using different basicity refining slags and [Al]_s_ contents during the smelting process. The melting points of CaO-SiO_2_-Al_2_O_3_ and MnO-SiO_2_-Al_2_O_3_ inclusions, in particular the latter, are relatively low and the liquid phase is likely to present at the soaking process, which is before the rolling process. In addition, the high Cr content (18 wt%) containing in 304 stainless steel is more likely to react with the liquid inclusions or solid–liquid mixes in the steel during the soaking process. This reaction will change the morphology, size, composition, and even crystal structure of the inclusions. Correspondingly, the mechanical properties of inclusions and their effects on the final products will be perhaps changed as well. Therefore, it is important to investigate the evolution behaviors of two main types of noninclusion during the soaking process.

Many researchers have noted that the nonmetallic inclusion would change in shape, size, and/or composition during heat treatment. Takahashi et al. [11] reported the inclusion in 18Cr-8Ni stainless steel would evolve from CaO-SiO_2_-Al_2_O_3_ to MnO-Cr_2_O_3_ during heat treatment at 800 °C to 1200 °C. Takano et al. [12] found that a large number of MnO-Cr_2_O_3_ inclusions precipitated during heat treatment and they could somehow pin austenite grain boundary in a 17Cr-9Ni stainless steel. Shibata et al. [13,14] discovered that inclusion transferred from MnO-SiO_2_ to MnO-Cr_2_O_3_ during heat treatment and they also discussed the effects of Si, Mn, Ni and Cr contents on the evolutions of inclusions. Taniguchi et al. [15] investigated the inclusion variation in martensitic stainless steel and proposed three steps for nonmetallic inclusion phase transformation during heat treatment. Ren et al. [16] also reported that the MnO-SiO_2_ type inclusion would transform to MnO-Cr_2_O_3_ spinel-type inclusion during heat treatment, and they proposed that the modification was as Cr reduced the SiO_2_ in SiO_2_-MnO-based inclusion.

Our previous work [17] has investigated the plasticized MnO-Al_2_O_3_-SiO_2_-based inclusion evolution during heat treatment and found that the chemical reaction between Cr and SiO_2_ as well as Ostwald ripening plays a significant role in the inclusion behavior during heat treatment. Those results indicate that behaviors of nonmetallic inclusion during the soaking process are still not fully understood. For example, the differences between two main types of inclusions, i.e., CaO-SiO_2_-Al_2_O_3_-based and MnO-SiO_2_-Al_2_O_3_-based during the industrial soaking process (soaking temperature and time are 1250 °C and 120 min, respectively) and their evolution mechanisms are rarely reported. Furthermore, the properties of the two types of inclusions after the soaking process are not clarified.

In the present study, two 304 steel slabs, containing mainly CaO-SiO_2_-Al_2_O_3_-based and MnO-SiO_2_-Al_2_O_3_-based inclusions, respectively, were manufactured and isothermal heat treatment for the different time at 1250 °C to reveal the nonmetallic inclusion transformation process during the industrial soaking process. Furthermore, a thermodynamic analysis was performed and Ostwald ripening was introduced to explain the inclusion evolution mechanism.

## 2. Materials and Methods 

**Figure 1:**
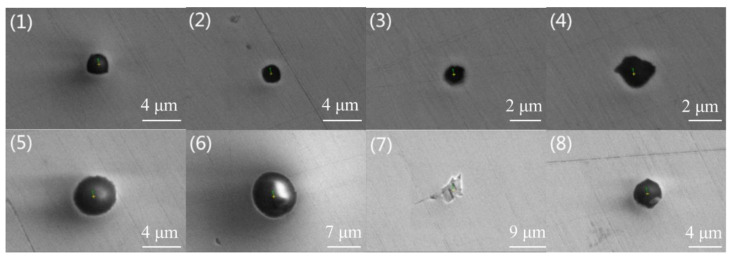
Typical morphologies of nonmetallic inclusions in the slab A. (**1**–**4**) small size inclusions; (**5**–**8**) large size inclusions.

**Figure 2:**
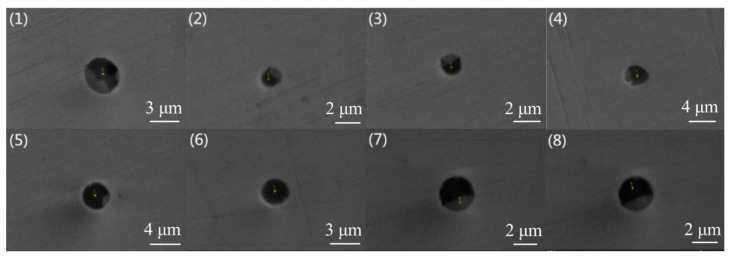
Typical morphologies of nonmetallic inclusions in the slab B. (**1**–**8**) small size inclusions (large size inclusions were not found).

The steel specimens were taken from two continuous casting slabs of industrial 304 stainless steel that were smelted by two basicity refining slags: steel A was smelted by a ladle furnace (LF) refining slag with basicity (CaO/SiO_2_) approximating 1.8 and corresponding refining slag basicity of steel B in LF was 1.5. The steel compositions and refining slag compositions were shown in Table 1 and Table 2, respectively. Moreover, the inclusions in the slabs of steel A and steel B were confirmed as mainly as CaO-SiO_2_-Al_2_O_3_-based and MnO-SiO_2_-Al_2_O_3_-based inclusion by scanning electron microscopy/energy-dispersive spectroscopy (SEM/EDS) before heat treatment, as shown in Figure 1 and Figure 2, respectively.

The steel specimens were machined into 15 mm × 15 mm × 15 mm cubic samples and were used for laboratory-scale isothermal heat-treated experiments. The heat treatment experiments were carried out in a muffle furnace with four MoSi_2_ elements (molybdenum silicide bar) in a 200 mL/min argon atmosphere. Firstly, the temperature was raised to 1250 °C by a heating rate of 10 K/min. Then, the temperature was held for 0, 30, 60 and 120 min for the steel samples marked as #1, #2, #3 and #4, respectively. The samples were quenched in the water after the heat treatment process. The quenched steel samples were ground and mirror polished for SEM-EDS observation. At the same time, inclusion analysis system equipped with SEM and EDS (EV018-INCAsteel, Oxford Instruments, Oxfordshire, UK) was applied to automatically analyse their morphology (i.e., aspect ratio), size and composition evolution during heat treatment on an analysed area of 5 mm × 5 mm. The working distance difference between the highest point and the lowest point of the sample detection area had been controlled to less than 5 μm. Otherwise it would exceed the auto-focus ability of the equipment. It should be pointed out that Fe and Ni in the inclusions detected by energy disperse spectroscopy (EDS) were removed to minimize the effects of steel matrix on inclusion composition, and some unclear spots were selected and excluded before the present inclusion analysis. In addition, the thermodynamic analysis was performed to study the inclusion transformation mechanism with the help of thermodynamic software FactSage 7.2 [18]. 

## 3. Results

Figure 1 and Figure 2 are typical inclusion morphologies in steel A and Steel B, respectively. In addition, their melting points calculated by the submodule *Equilib* of FactSage 7.2 according to their compositions, are also listed in the Table 3 and Table 4, respectively. The morphologies of inclusions in the two steel samples are mainly spherical, and their sizes are mainly lower than 5 μm. However, the inclusion components and their corresponding melting points are significantly different. As shown in the Table 3, the inclusions in steel A are mainly composed of CaO, SiO_2_ and Al_2_O_3,_ as well as small amounts of MnO, MgO and/or TiO_2_. Calculated by FactSage 7.2, the melting points of those inclusions are between 1391 °C to 2050 °C, and the average melting point is 1561 °C, which is much higher than the open-rolling temperature region (i.e., 1150–1250 °C).

Compared with the inclusions in steel A, CaO contents of the inclusions in steel B are much less while the MnO contents are much more. Furthermore, the Al_2_O_3_ contents in the inclusions are much less as well. The melting points of the inclusions in steel B are between 1143 °C to 1479 °C, and their average is 1238 °C that is close to the open-rolling temperature region, indicating the inclusions are well plasticized since they are likely to soften at the rolling temperature. In a word, the inclusion in steel A is CaO-SiO_2_-Al_2_O_3_-based, and have a relative high melting point due to being smelted by the high basicity refining slag. The inclusion in steel B is plasticized MnO-SiO_2_-Al_2_O_3_-based, and have a low melting point due to the low basicity slag refining.

**Figure 3:**
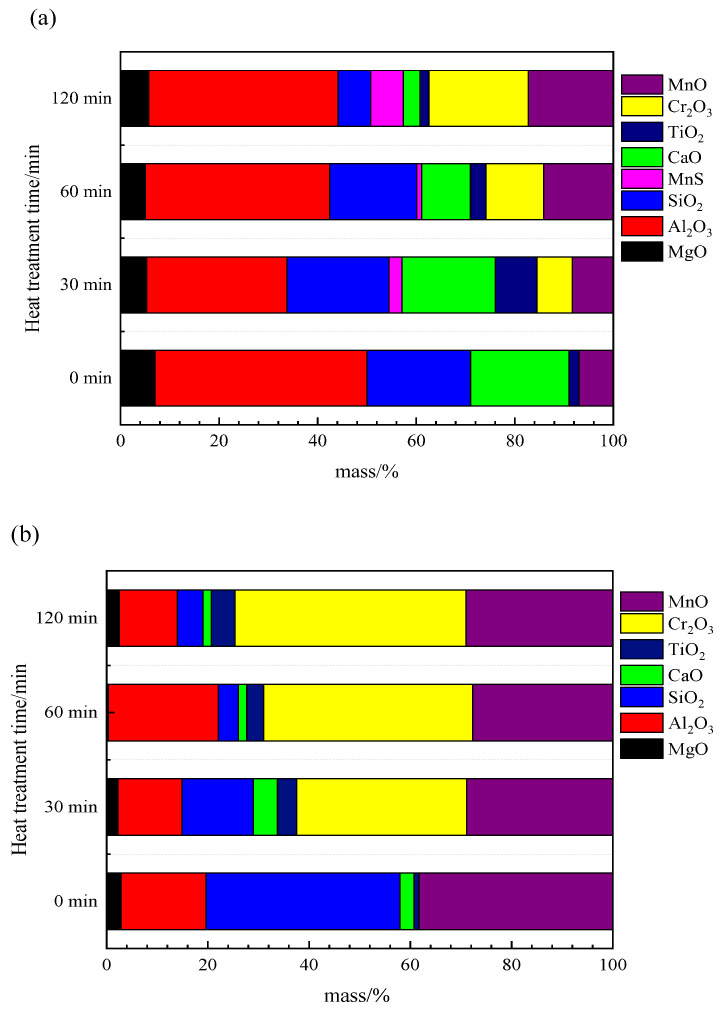
Variations in the average composition of inclusions during heat treatment: (**a**) steel A, (**b**) steel B.

Figure 3a,b show the average inclusion composition evolution during the heat treatment process of steel A and steel B, respectively. For the steel A as shown in Figure 3a, the average Cr_2_O_3_ and MnO contents gradually increase with increasing heat treatment time. In contrast, the CaO and SiO_2_ contents decrease significantly. The contents of Al_2_O_3_ and MgO are somewhat fluctuant, and do not show obvious variation during heat treatment. As shown in Figure 3b, the change of each component is similar to that of Figure 3a. However, the average Cr_2_O_3_ content of inclusion in steel B increases much more than that in steel A, while the SiO_2_ content decreases significantly along with the heat treatment time. In a word, during heat treatment, Cr_2_O_3_ and MnO of the inclusions in the two samples both increase while the SiO_2_ and CaO contents decrease. However, there are also some differences between the inclusion composition evolution in the two steel: Cr_2_O_3_ in steel B increases much more than that in steel A; the SiO_2_ content in steel B decreases more obviously than in steel A.

**Figure 4:**
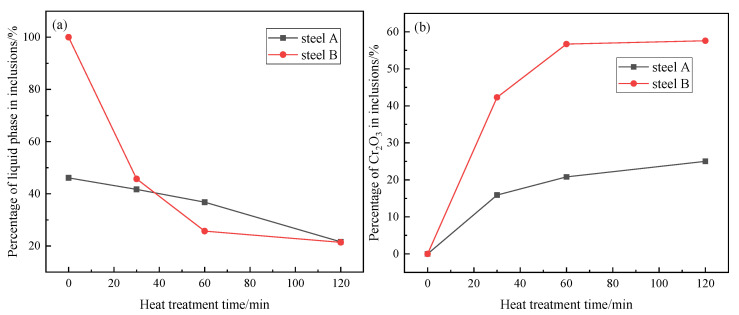
Change of the liquid phase (**a**) and Cr_2_O_3_ (**b**) content in the inclusion of steel A and steel B with the heat treatment time at 1250 °C calculated by FactSage 7.2.

Figure 4 shows the variations of the liquid phase and Cr_2_O_3_ content in the inclusion of steel A and steel B with the heat treatment time at 1250 °C calculated by FactSage 7.2 based on the average composition of inclusion. With the Cr_2_O_3_ content increase during heat treatment, MnO-Cr_2_O_3_ spinel phase in the inclusion increase correspondingly. The MnO-Cr_2_O_3_ spinel phase in the inclusion of steel B increases much rapidly than that in steel A. In addition, the percentage of the liquid phase in steel A and steel B decreases with increasing heat treatment time, which is due to the change of inclusion composition. Associated with the aforementioned phenomena (Figure 3 and Figure 4), it is therefore indicated that MnO-Cr_2_O_3_ spinel continuously precipitates during heat treatment.

**Figure 5:**
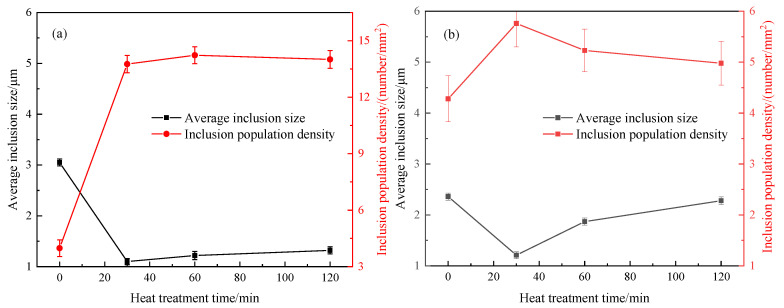
Variations in the population density and average size of inclusions at 1250 °C in steel A (**a**) and steel B (**b**).

Figure 5a,b show the changes of inclusion population density and average inclusion size during heat treatment in steel A and steel B, respectively. Interestingly, the above indexes in steel A and steel B show a similar tendency. After heat treatment for 30 min, the inclusion population density increases as many small size Cr_2_O_3_-MnO-based inclusions precipitate during the heat treatment process, and the average inclusion size decreases correspondingly. With increasing heat treatment time, the inclusion population density decreases, and their average size gradually increases. This is because the inclusion growth follows the Ostwald ripening during heat treatment: the small inclusions dissolve and the larger inclusions grow. Ren et al. [16] also observed a similar inclusion number evolution and size distribution, which is consistent with the findings in Figure 5. Those phenomena in the Figure 5 are more popular for inclusion transformation during heat treatment. Compared with steel A, steel B has had an even more volatile ride after 30 min due to the higher transformation speed caused by lower melting points.

**Figure 6:**
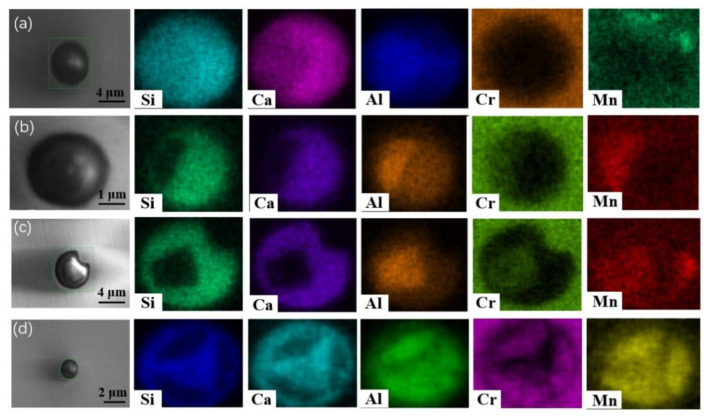
SEM-mapping of some typical inclusions after heat treatment for different time in steel A: (**a**) #1, 0 min; (**b**) #2, 30min; (**c**) #3, 60 min and (**d**) #4,120 min.

**Figure 7:**
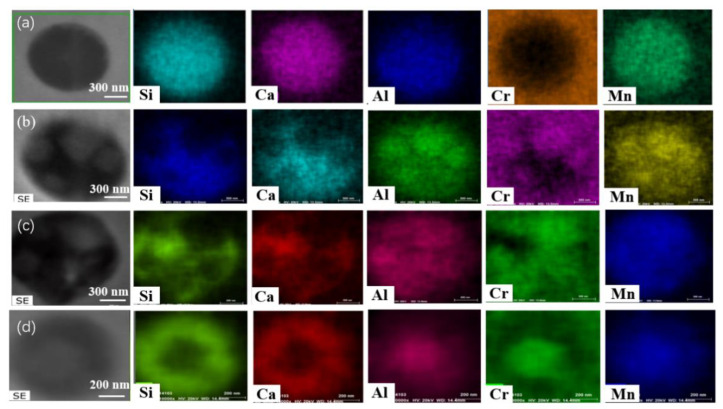
SEM-mapping of some typical inclusions after heat treatment for different time in steel B: (**a**) #1, 0 min; (**b**) #2, 30min; (**c**) #3, 60 min and (**d**) #4,120 min.

Figure 6 and Figure 7 show SEM-mapping images of some typical inclusions in the steel samples at different stages of the heat treatment process. At the beginning of the heat treatment as shown in Figure 6a and Figure 7a, no Cr oxide-concentrated regions were found within all inclusions. The differences are that: (1) there is a triangular Al-concentrated region in the left part of inclusion of Figure 6a while Al distributes homogeneously within the inclusion in Figure 7a; and (2) very little Mn was detected in the inclusion in Figure 6a, while much more Mn content was detected uniformly distributed in the inclusion in Figure 7a. As shown in Figure 6b and Figure 7b, some quadrate Cr_2_O_3_-MnO-Al_2_O_3_-concentrated regions were observed in the outer layer within the inclusion, and the other parts are CaO-SiO_2_ became enriched in the composition after heat treatment for 30 min. However, the inclusions in Figure 6c and Figure 7c are different: a faceted Cr_2_O_3_-MnO-Al_2_O_3_-concentrated core with a light color in the center of inclusion listed in Figure 6c, while most of inclusions were Cr_2_O_3_-MnO-concentrated region in the outer layer and only a small CaO-SiO_2_-concentrated region in the top left corner of inclusion in Figure 7c. Interestingly, some Cr_2_O_3_-MnO-Al_2_O_3-conce_ntrated regions were inserted in the inclusion in steel A after heat treatment for 120 min as shown in Figure 6d. In the contrast, a Cr_2_O_3_-MnO-Al_2_O_3-_concentrated core wrapped by a ring-like CaO-SiO_2_-concentrated region was observed in the inclusion of steel B after heat treatment for 120 min as shown in Figure 7d. In short, there are some interesting similar behaviors of the inclusions evolution during heat treatment, although the compositions at the beginning are quite different. The Cr_2_O_3_ contents in the inclusions are increasing with increasing heat treatment time, but the Cr element does not always diffusion from the outer layer to the inner part; Cr_2_O_3_-MnO or Cr_2_O_3_-MnO-Al_2_O_3-_enriched regions and the CaO-SiO_2_-concentrated region seem incompatible and complementary among the inclusions in most inclusions in the two steels. The difference is that the Mn element concentration of inclusion in steel A is much less than that of inclusions in steel B, which agrees with the inclusion composition in Figure 3. 

## 4. Discussion

Thermodynamic calculated results (Figure 4) and the experimental results show a large number of Cr_2_O_3_-MnO tetragonal spinel contained some amount of Al_2_O_3_ would precipitate during heat treatment process. They result in the change of inclusion composition as shown in Figure 3 and inclusion size and density, as shown in Figure 5. 

According to the density functional theory calculations [19,20], the solute atoms can enter the oxide defect and produce a mixed oxide structure, which indicates that Cr atoms may have a chemical reaction with oxygen in inclusions. Some researchers [14,16] developed the idea that the formation of MnCr_2_O_4_ spinel during the heat treatment process is due to the reaction between Cr in the solid steel matrix and 2MnO·SiO_2_-type inclusion, as shown in Equation (1).

(1) $$2\left[\mathrm{Cr}\right]+2\mathrm{MnO}\cdot {\mathrm{SiO}}_{2}=\mathrm{MnO}\cdot {\mathrm{Cr}}_{2}O_{3}+\left[\mathrm{Mn}\right]+2\left[\mathrm{Si}\right]$$

In fact, the Cr in steel matrix has the possibilities to react with MnO, SiO_2_, Al_2_O_3_ and even CaO, thus the thermodynamic analysis among these chemical reactions should be performed to understand the inclusion evolution mechanism.

The reaction between [Cr] and (MnO) and its standard Gibbs energy $\Delta G_{}^{\theta }$ are expressed as Equations (2) and (3) [21]. Equation (4) is the Gibbs free energy $\Delta G_{(\mathrm{MnO})}$ of the actual reaction.

(2) $$2[\mathrm{Gr}]+3(\mathrm{MnO})=({\mathrm{Cr}}_{2}O_{3})+3[\mathrm{Mn}]$$

(3) $$\Delta G^{\theta }=-84310+40.035T$$

(4) $$\Delta G_{\left(\mathrm{MnO}\right)}=\Delta G^{\theta }+RT\ln(\frac{a_{[\mathrm{Mn}]}^{3}a_{{\mathrm{Cr}}_{2}O_{3}}}{a_{\left[\mathrm{Cr}\right]}^{2}a_{\mathrm{MnO}}})$$

where *R* is gas constant, 8.314 J/(mol·K); *T* represents temperature (i.e., 1523 K); $a_{\left[\mathrm{Mn}\right]}$ and $a_{\left[\mathrm{Cr}\right]}$ are the element activity in steel matrix relative to 1% standard state; $a_{\mathrm{MnO}}$ and indicate the MnO and Cr_2_O_3_ activities in the inclusion. The Al_2_O_3_-SiO_2_-MnO inclusions are in liquid phase, or most of them are in liquid at 1523 K; thus the activity of the MnO and Cr_2_O_3_ are calculated by applying of the thermodynamic software FactSage 7.2 [18].

Similarly, the Gibbs free energies of the other reactions can be obtained according to their corresponding standard Gibbs free energy as shown in Table 5. Table 6 shows the average contents of the two typical inclusions and the corresponding activities calculated by FactSage 7.2.

The activity coefficients $f_{\mathrm{Cr}}$ and $f_{\mathrm{Mn}}$ are calculated by the Wagner formula in Equation (5), where $e_{i}^{j}$ is the first-order activity interaction coefficient of elements *j* to *i* relative to the diluted solution. These values are from reference [22]. It should be pointed out that the activity interaction coefficients applied in the present calculations are selected from those measured based on Fe-Cr-Ni stainless steel hot metal, or those are confirmed applicable for the 18 pct Cr-8 pct Ni stainless steel. In addition, the second interaction coefficients of Cr and Ni to the elements in steel are performed because of high Cr and Ni content in 18 pct Cr-8 pct Ni stainless steel [1,23,24,25].

(5) $$\mathrm{lg}f_{i}=\sum_{}^{}e_{i}^{j}[\mathrm{mass}\%j]+r_{i}^{\mathrm{Cr}}{\left[\mathrm{Cr}\%\right]}^{2}+r_{i}^{\mathrm{Ni}}{\left[\mathrm{Ni}\%\right]}^{2}+2r_{i}^{\mathrm{Cr},\mathrm{Ni}}[\mathrm{Cr}\%]{\left[\mathrm{Ni}\%\right]}^{2}$$

**Figure 8:**
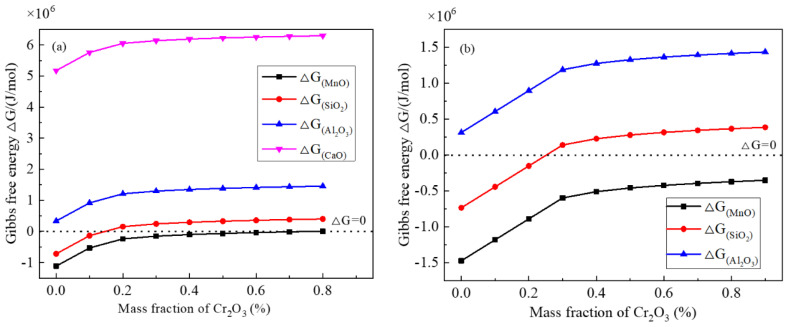
Gibbs free energy changes of some typical possible chemical reactions vs different Cr_2_O_3_ contents in inclusion at soaking temperature 1250 °C: (**a**) steel A, (**b**) steel B.

Figure 8a,b show the Gibbs free energy change of the foregoing typical chemical reactions with different Cr_2_O_3_ contents in inclusions at 1250 °C. In the two figures, with increasing Cr_2_O_3_ content, the Gibbs free energy increases, and finally are positive, indicating it is more and more difficult to take place for these chemical reactions. The Gibbs free energy for the reaction between Cr and MnO is lower than 0, even if a larger Cr_2_O_3_ content in an inclusion. Moreover, the Gibbs free energy between Cr and SiO_2_ is larger than 0 in the case of the amount of Cr_2_O_3_ content larger than 20 mass%. For the reaction between Cr and Al_2_O_3_ or CaO, the change of free Gibbs energy is almost positive even if very low Cr_2_O_3_ content in an inclusion. Hence, Cr is most likely to reduce the MnO in the inclusion, followed by the SiO_2_, but Al_2_O_3_ or CaO in the inclusion is not likely to be reduced by Cr during heat treatment process. Compared with steel A, inclusions in steel B are more likely to react with Cr, which accords with the changes of Cr_2_O_3_ in Figure 3.

It is true that some phenomena can be explained by chemical reactions. However, MnO content increases continuously or is almost not reduced obviously, while SiO_2_ and even CaO contents are reduced, as shown in Figure 3. Obviously, this is not in agreement with the thermodynamic results as shown in Figure 8. In addition, if MnO-Cr_2_O_3_ inclusion is formed due to the chemical reaction Equation (1), the inclusion size would be almost constant but inclusion diameter is changed a lot in present experimental results (Figure 5) as well as in other researchers’ results [14,16]. Therefore, it can be deduced that there is another driving force to cause inclusion transformation during heat treatment including chemical reactions between Cr and inclusions. 

Ostwald ripening is a very common phenomenon for the secondary phase growth during heat treatment. It is characterized by that the smaller inclusions dissolve, and the larger inclusions grow by absorbing the smaller inclusions during heat treatment. The present observations, such as inclusion size and their population density evolution as shown in Figure 5 and some SEM-mapping images in Figure 6 and Figure 7, accord with the features of Ostwald ripening very well. Therefore, the evolutions of some inclusions during heat treatment mainly follow the rule of Ostwald ripening rather than the chemical reactions.

According the classical Ostwald ripening [26], the interface energy generates with the secondary phase precipitating during heat treatment, and total interface energy would generate when the inclusion with a smaller radius. The driving force of Ostwald ripening is the difference of interface energy between steel matrix and smaller size inclusion and between matrix and larger size inclusion.

**Figure 9:**
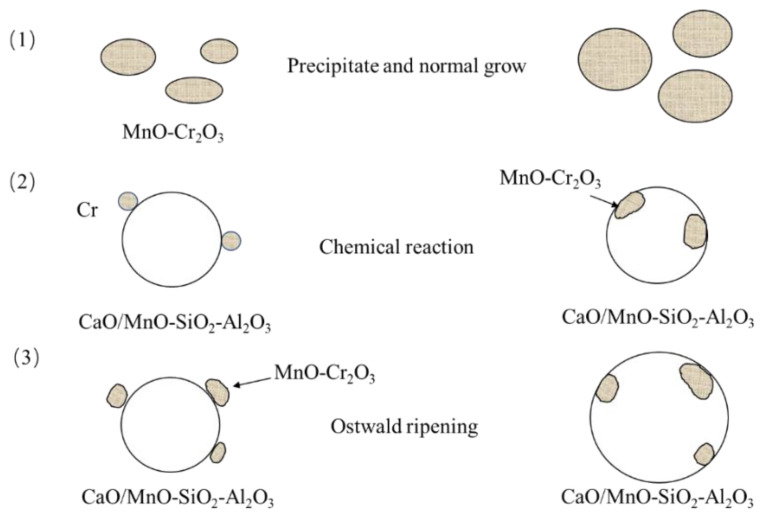
Schematic diagram of three possible evolution processes of the inclusion during the soaking process.

In summary, according to the experimental observation and thermodynamic analysis, there are three steps for inclusion evolution during the heat treatment process as schematically shown in Figure 9: (1) MnO-Cr_2_O_3_ spinel particles precipitate and normal grow; (2) Chemical reactions between Cr and CaO/MnO-SiO_2_-Al_2_O_3_ inclusion; (3) CaO/MnO-SiO_2_-Al_2_O_3_ inclusions and MnO-Cr_2_O_3_ inclusions grow by Ostwald ripening, namely by absorbing smaller MnO-Cr_2_O_3_ particles. 

Three steps always take place simultaneously during the heat treatment process, in particular the later two steps are even likely to occur on the same one inclusion. Thus, it is difficult to distinguish quantitively, for which the step is the dominant mechanism for inclusion evolution during the heat treatment process, which is needed further investigated. But as shown in Figure 3, average MnO contents increase or keep almost unchanged which is, obviously, not the effects of the chemical reactions, thus it could conclude that the Ostwald ripening play a very important role on the inclusion modification during heat treatment process.

## 5. Conclusions

Transformations of two common types of nonmetallic inclusion in 304 stainless steel, smelted by high basicity refining slag and low basicity slag, respectively, were investigated in laboratory-scale furnace at 1250 °C. The following conclusions were obtained.

Firstly, the slag system with high basicity of LF results in the formation of CaO-SiO_2_-Al_2_O_3_ inclusions with a high melting point. Moreover, MnO-SiO_2_-Al_2_O_3_ inclusions are mainly formed under slag with low basicity, which are almost in the liquid phase at the heat treatment temperature before rolling.

Secondly, inclusion population density increases at the first stage and then decreases, and their average size firstly decreases and then increases due to a large number of Cr_2_O_3_-MnO particles precipitating and their growth during heat treatment process. 

Thirdly, there is almost no Cr_2_O_3_ before the heat treatment, but Cr_2_O_3_ precipitates gradually increase along with the heat treatment process. The increasing rate of Cr_2_O_3_ content in MnO-SiO_2_-Al_2_O_3_ inclusion is much higher due to its low melting point. The increasing of Cr_2_O_3_ content in the inclusion would increase their melting points and lower their plasticities.

Finally, both experimental results and thermodynamic analysis show that there are three steps for inclusion evolution during the heat treatment process: (1) many small size MnO-Cr_2_O_3_ spinel particles precipitate and grow; (2) Chemical reactions between Cr and CaO/MnO-SiO_2_-Al_2_O_3_ inclusion; (3) by Ostwald ripening, namely CaO/MnO-SiO_2_-Al_2_O_3_ inclusions and MnO-Cr_2_O_3_ inclusions grow by absorbing smaller MnO-Cr_2_O_3_ particles. Ostwald ripening plays an important role on inclusion evolution during the soaking process.

## Figures and Tables

**Table 1:** 304 stainless steel composition in the present experiment, mass %.

No.	C	Si	Mn	P	S	Cr	Ni	Als/ppm	T.O/ppm
A	0.048	0.415	1.164	0.032	0.0015	17.98	8.17	19	33
B	0.047	0.431	1.204	0.032	0.0012	18.15	8.01	9	35

Note: T.O represents total oxygen in stainless steel samples.

**Table 2:** Top slag composition at the finial LF refining in the present experiment, mass %.

No.	CaO	SiO_2_	Al_2_O_3_	MgO	CaF_2_	Cr_2_O_3_	TiO_2_	MnO	FeO
A	45.9	26.2	2.9	6.7	16.7	0.10	0.49	0.07	0.08
B	46.4	30.3	2.1	7.7	12.4	0.27	0.31	0.32	0.26

**Table 3:** Compositions of typical inclusions in Figure 1.

NO.	CaO/%	SiO_2_/%	MgO/%	Al_2_O_3_/%	MnO/%	CaS/%	TiO_2_/%	D/μm	Tf/℃
1	3	3	15	61	16	0	2	2.00	1651
2	26	28	6	30	5	0	4	1.20	1458
3	24	29	7	35	4	0	0	1.24	1511
4	20	21	4	35	17	0	4	2.61	1496
5	21	25	10	34	5	0	5	4.27	1523
6	41	29	9	19	1	0	0	9.80	1414
7	0	0	0	100	0	0	0	10.00	2050
8	23	31	5	27	11	3	0	3.48	1391
Average	20	21	7	43	7	0	2	4.33	1561

Note: D presents inclusion diameter and T_f_ means the melting point of the inclusion.

**Table 4:** Compositions of typical inclusions in Figure 2.

NO.	CaO/%	SiO_2_/%	MgO/%	Al_2_O_3_/%	MnO/%	CaS/%	TiO_2_/%	D/μm	Tf/℃
1	0	46	0	7	44	4	0	3.00	1213
2	0	50	0	19	31	0	0	1.25	1172
3	0	42	0	15	40	0	3	1.43	1157
4	10	40	16	22	12	0	0	2.96	1363
5	5	45	0	21	29	0	0	2.80	1142
6	9	44	7	19	22	0	0	2.75	1214
7	0	17	0	26	46	0	12	2.36	1479
8	3	42	0	19	36	0	0	2.36	1167
Average	3	41	3	18	32	0	1	2.36	1238

**Table 5:** Some possible reactions and their standard Gibbs free energy.

Chemical reaction	Standard Gibbs free energy	Reference
$2\left[\mathrm{Cr}\right]+\frac{3}{2}\left({\mathrm{SiO}}_{2}\right){=\mathrm{Cr}}_{2}O_{3}+\frac{3}{2}\left[\mathrm{Si}\right]$	57350+30.525T	[21]
$2\left[\mathrm{Cr}\right]{+(\mathrm{Al}}_{2}O_{3}{)=\mathrm{Cr}}_{2}O_{3}+2\left[\mathrm{Al}\right]$	417690−35.975T	[21]
$2\left[\mathrm{Cr}\right]+3\left(\mathrm{CaO}\right){=\mathrm{Cr}}_{2}O_{3}+3\left[\mathrm{Ca}\right]$	170690+357.825T	[21]

**Table 6:** Average inclusion composition and the calculated activities of two typical inclusions at 1250 °C.

SteelA			Steel B	
Composition	Content/wt%	Acticity α	Content/wt%	Acticity α
Al_2_O_3_	40.8	0.215	16.9	0.057
SiO_2_	29.6	0.204	39.5	0.280
MnO	16.3	0.082	43.6	0.210
CaO	13.3	0.001	-	-
